# Coastline Levels of Dissolved Heavy Metals in the Estuarine Water–System of Vigo

**DOI:** 10.3390/ijerph18042136

**Published:** 2021-02-22

**Authors:** Benita Pérez-Cid, Elena Falqué, Jesus Simal-Gandara

**Affiliations:** 1 Analytical Chemistry Group, Department of Analytical and Food Chemistry, Faculty of Chemistry, University of Vigo–Vigo Campus, E36310 Vigo, Spain; benita@uvigo.es; 2 Analytical Chemistry Group, Department of Analytical and Food Chemistry, Faculty of Science, University of Vigo–Ourense Campus, E32004 Ourense, Spain; efalque@uvigo.es; 3 Nutrition and Bromatology Group, Department of Analytical and Food Chemistry, Faculty of Science, University of Vigo–Ourense Campus, E32004 Ourense, Spain

**Keywords:** maritime traffic, dissolved trace metals and metalloids, estuarine-coastal water-system, river influence, Vigo Ría, summer

## Abstract

Limited attention has been directed toward the effects of maritime traffic on heavy metals and metalloids in seawater. Water samples were collected from the estuary of Vigo Ría in the summer of 2018. The chemical distribution of ten dissolved trace metals and metalloids (Cr, Mn, Fe, Cu, Zn, As, Se, Cd, Hg, and Pb) in water was determined from north to south (where the biggest city in the region is) and from east to west (where the maritime traffic is higher). Metal concentration in waters showed that most metals were below recommended water quality criteria by the United States Environmental Protection Agency (EPA). One of the samples had a Cu concentration higher at the entrance of the Vigo estuary, where maritime traffic is very important. Cu and Zn concentrations were highly correlated between them, showing a similar pollution origin, probably due to anti-fouling paints. Mn and Fe are elements influenced by river sources.

## 1. Introduction

The presence of heavy metals in the environment can occur naturally or as a result of anthropogenic activities located in developed areas. It is well known that industrial acti-vity has caused an elevated accumulation of heavy metals in zones with high rates of industrialization [1]. In the aquatic systems, heavy metals are usually distributed between the aquatic medium and the suspended particle matter and sediments, thereby resulting in considerably higher levels of heavy metals found in sediments than in the water co-lumn. This fact can be attributed to the easy interaction between metals and suspended particle matter [2], which is eventually incorporated into the fine sediment [3]. On the other hand, some elements, such as Fe and Mn, easily participate in redox processes in the aqueous medium and can form hydroxides and oxides or even precipitate as sulphides. Consequently, they can also be removed from the sediment fraction. In a previous work focused on sediments of the Vigo Ría [4], it was observed that more than 65% of total Fe and Mn contents were found in the residual fraction of the sediment. In other research based on evaluated dissolved forms of Fe and Mn in the water column of the Black Sea, it was observed that the concentration of both elements increased at depths in which oxygen consumption rose and hydrogen sulphide appeared [5]. Therefore, in aquatic environments, heavy metals are mainly accumulated in sediments, with concentrations several orders of magnitude higher than those found in adjacent waters.

**Figure 1:**
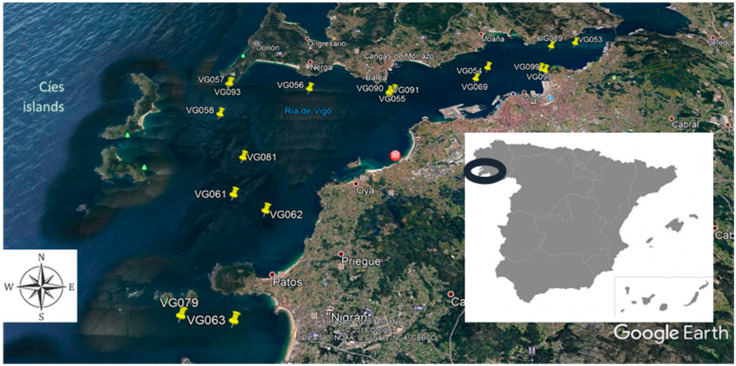
Sampling points along the Vigo Ría in summer 2018.

Galician Rías are estuarine-coastal systems located in the northwest of the Iberian Peninsula and constitute a reference in the study of heavy-metal pollution on the Atlantic Coast. In particular, the Vigo Ría (Figure 1), which has a small river flowing into it, receives important anthropogenic contributions due to the high industrial activity located around Vigo town, including shipyards, an automobile factory, canning industry, and aquaculture [6]. The Vigo estuary is the deepest and southernmost of the Rías Baixas in Galicia (Spain). It is located in the south of the province of Pontevedra, and it is V-shaped with a length of over 33 km and 156 km^2^ of total surface [7]. It is an open system with a major exchange, oceanic waters, and its central axis oriented NE–SW (Figure 1). Partially closed by the Cíes Islands, which are part of the Atlantic Islands National Park, the estuary’s maximum width (5.2 km wide, 53 m deep) is at the mouth of the embayment and diminishes progressively (600 m wide, 7 m deep) at the Rande Strait [8,9].

The location of Vigo Ría at the northernmost limit of the Eastern North Atlantic upwelling system promotes its biodiversity and ecological richness during the spring and summer when the northerly winds along the coast transport surface water offshore, which is replaced by a cold, nutrient-rich, deep water mass called Eastern North Atlantic Central Water [10]. Historically, the Vigo estuary has been a favorable area for fishing and shell-fishing for human consumption; therefore, it is important to highlight the need to control the level of contamination in this area. The catchment (578.2 km^2^) is heavily urbanized and industrialized (>21%) [11], and activities include shipbuilding, canning, and automobile and steel manufacturing. Metal pollution is restricted to the inner estuary as a result of urban and industrial discharges and because of intense activity of the Port of Vigo with chronic Pb pollution due to discharge from a ceramic factory located at the head of the estuary [12]. Other sources are natural, related to catchment and upwelling processes. Mariculture rafts in the northern estuary have influenced the distribution of metals by increasing the carbon content and decreasing grain size producing metal sinks. Tidal currents act to redistribute metals from accumulation zones. In previous studies [13,14,15,16], an enrichment of some trace metals in sediments of the Vigo Ría was observed; however, the available information about dissolved trace metals is much more limited [7,17,18,19], probably due to their low concentration found in seawater and the difficulties associated with their quantification in this water matrix.

Heavy metals are among the highest serious pollutants of water ecosystems because of their high possibility of entering and being accumulated in the nourishment chain [20]. Heavy metals pollution sources include the runoff from agricultural and urban areas; discharges from factories and municipal sewer systems; activities in the harbors; leaching from dumps and former industrial sites; and atmospheric deposition [21]. Possible effects from heavy metals are commonly circumscribed to locations closer to major cities or industrialized areas on the coastal margin and spot-draining areas of strenuous agriculture, together with maritime traffic [22].

By taking the above into account, the main aim of this work was to evaluate the content of dissolved trace metals and metalloids (Cr, Mn, Fe, Cu, Zn, As, Se, Cd, Hg, and Pb) in different sampling points of the Ría of Vigo to evaluate the possible existence of important pollution sources in the studied area. In addition, the data obtained were statistically analyzed and compared with other results reported by previous studies, with the aim to assess the current state of contamination of the estuary.

## 2. Materials and Methods

### 2.1. Water Sampling and Monitoring Campaign

In July 2018, a campaign was carried out in the Vigo estuary to the European CoastObs project [23]. A total of 18 sampling points (Supplementary Table S1) were selected along the Vigo estuary (Figure 1), from the Cíes Islands to the Rande Strait, in order to obtain a complete report about the possible existence of pollution sources in the studied area. In these points, seawater samples of about 700 mL were collected in metal-free polyethylene plastic bottles at a depth of 4 m using a positive crankcase ventilation (PCV) hose from the sampling stations and transported in a cooler. Fifty milliliters were intended for metals and metalloids analysis, and the rest was meant for other researchers to monitor harmful algal blooms [23] and drug residues [24].

### 2.2. Metal and Metalloids Detection and Determination

In the laboratory, for determination of the dissolved metals and metalloids in the seawaters, the samples were filtered through a membrane filter with a pore diameter of 0.45 μm. A portion of the sample was used to rinse the filter flask before being discarded, and then 5 mL of the sample was filtered. The filtrate was immediately acidified with 0.1 mL nitric acid (69% Hiperpur, Panreac, Barcelona, Spain) (1/1, v/v) and stored at 4 ℃ until its analysis the next day. Glassware used in the analysis may contain contamination of trace metals; therefore, bottles and pipette tips were soaked in nitric acid for one day and then rinsed with deionized water to remove trace-metal impurities from the glassware. Ultrapure Milli-Q (Millipore, Bedford, MA, USA) water was used for all operations, namely, cleaning, rinsing, preparation, and dilution of solutions. The different standard solutions, as well as blank solutions, were prepared using the same container and acid as the water samples.

Metal and metalloid elements of 18 seawater samples were analyzed in triplicate using an inductively coupled plasma Agilent 7700x-model mass spectrometer (ICP-MS; Agi-lent Technologies, Santa Clara, CA, USA) quadrupole [25] with a sample introduction system consisting of a Micromist glass low-flow nebulizer, a double-pass glass spray chamber with a Peltier system (3 ℃), and a quartz torch. The instrumental measurement parameters in the ICP-MS plasma were radio frequency power (W): 1550; sample flow (L min^−1^): 0.6; and argon flow (L min^−1^): refrigerant at 15.0, auxiliary at 0.59, and nebulizer at 0.4. Standard solutions for calibrations were made with five concentrations obtained by diluting a commercial standard multielement reagent VI (Merck, Darmstadt, Germny) with deionized water (Table 1). ^103^Rh and ^159^Tb were used as internal standards, adding a well-known concentration to both samples and targets. The isotopes used to quantify the studied elements were ^52^Cr, ^55^Mn, ^56^Fe, ^63Cu^, ^66Zn^, ^75^As, ^78^Se, ^111^Cd, ^202^Hg, and ^208^Pb.

To verify that the spectral interferences were effectively eliminated or corrected, a study was carried out using synthetic solutions and known concentrations of the elements that make up the interfering compounds. Accordingly, synthetic seawater was used to prepare a sample enriched with known contents of most of the elements studied in this work. The results obtained, expressed as mean value and standard deviation of three determinations, are shown in Supplementary Table S2. In all cases, the experimental results were in good agreement with the spiked contents, reaching recovery percentages between 92.31 and 102.2%. Furthermore, the statistical comparison of values by *t*-test, at a confidence level of 95%, indicates that no significant differences were found. These results allowed us to verify the analytical quality of the results reported in this work, including the correction of possible polyatomic interferences caused by the sample matrix in the argon plasma.

### 2.3. Statistical Analysis

Statistical analyses were performed using Statgraphics Centurion XVI v. 16.1.11 for Windows (Statgraphics Technologies Inc., Plains, Virginia, USA). The statistically significant differences between group means were estimated by parametric statistics (analysis of variance, ANOVA) and least significant difference tests at the 95% probability level.

## 3. Results and Discussion

**Figure 2:**
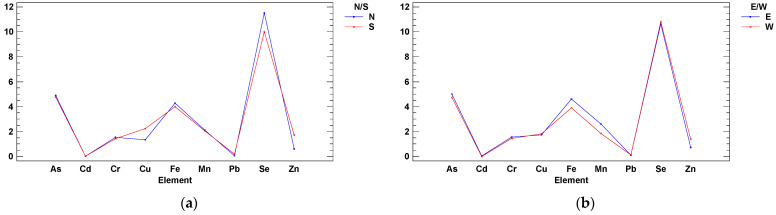
Two-way ANOVA interactions amongst elements and cardinal axis {level at µg L^−1^ (*y* axis) vs. elements (*x* axis)}: (**a**) N/S, with Cu and Zn higher levels in the south (Vigo harbor) and (**b**) E/W, with Mn and Fe higher levels in the east (inner estuary).

**Figure 3:**
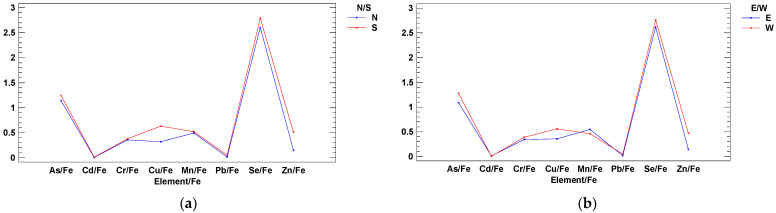
Two-way ANOVA interactions amongst element ratios vs. Fe and cardinal axis (element level/Fe level ratios (*y* axis) vs. elements (*x* axis)): (**a**) N/S, with Cu/Fe & Zn/Fe higher levels in the south (Vigo harbor) and (**b**) E/W, with Cu/Fe and Zn/Fe higher levels in the west (outer estuary).

The study area was divided into four quadrants/locations (southwest, northwest, southeast, and northeast) centered on the geometric center of the sampled area (Figure 1 and Supplementary Table S1). Multifactor two-way ANOVA for concentration levels was performed, and two significant interactions were detected (Elements by N/S areas and Elements by W/E areas) (Figure 2). The same analysis was repeated but normalizing all elements against Fe level (Figure 3). In these cases, the average plus confidence interval is calculated for the grouping formed by the two interacting variables in any of the two interaction plots.

**Figure 4:**
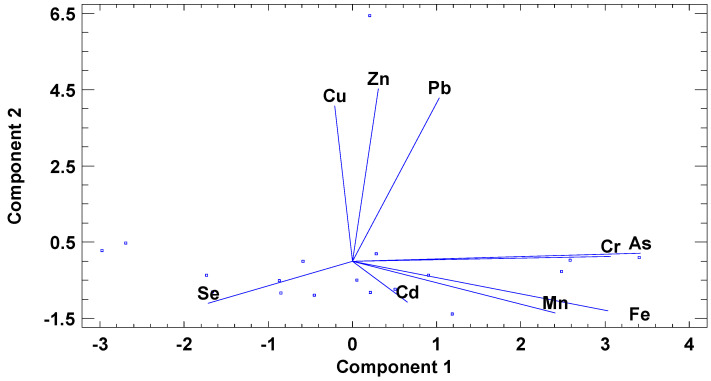
Biplot of the two main components obtained by principal component analysis (PCA) representing both water samples and detected elements.

Basic and descriptive statistics (mean ± standard deviation, minimum and maximum values, median ± interquartile range, and lower and upper quartiles; Table 2), together with linear regression analysis between Cu (dependent) and the rest of the elements (independent variables) were also performed. With principal component analysis (PCA) and varimax rotation, it was possible to separate the water samples by their content in the chemical parameters in a two-dimensional space and find correlations amongst variables (Figure 4).

In Vigo Ría in summer, Se >> As = Fe > Cu = Zn = Mn = Cr >> Cd = Pb (Figure 2), whereas Hg was not detected. Considering differences by location areas, Cu and Zn were higher in the south of the estuary (by the city harbor of Vigo; Figure 2a), and Mn and Fe were higher in the east of the estuary, in its inner part next to the river end (Figure 2b). The normalization of metal data by the contents of a conservative element such as Al, Fe, Li, Rb, Sc, and organic carbon, which represents one or more major metal carriers of sediments (e.g., clay minerals, iron, and manganese hydroxides, oxides, and organic matter, etc.), is quite a useful approach (Al was used by Ho et al. [26]; Fe was used by García et al. [15]. If we normalize metal levels vs. Fe as a natural source tracer, then the Cu/Fe and Zn/Fe ratios are higher in the area of influence of the Vigo city harbor, the south side and the west side at the exit of the estuary (Figure 3). These two elements are mainly associated with antifouling boat paintings [27].

Marine sediment samples analyzed by Monaco et al. [28] presented metal contamination (Cd, Cu, Pb, and Zn), mainly in the harbor and industrial and commercial maritime areas. Cu and Zn were also highly correlated, and Pb (also associated with paintings) was correlated with them (Figure 4). Sediments at port sites were clearly enriched with metals compared to the other sampling sites; this is especially remarkable for Cu and Zn [29]. Fe was correlated with Mn, which is indicative of a natural origin, associated with Fe-Mn oxides [30]. The almost undetectable levels of Cd were correlated to Fe and Mn too. Moreover, As was positively correlated with Cr and negatively correlated with Se, indicating different sources for As and Se. Sources of As and Cr contamination are predominately associated with anthropogenic activities arising from the applications of agriculture fertilizers and pesticides, disposal of industrial wastes, sewage discharge, and combustion of fuels [3]. After eight sampling campaigns (2000–2010), Besada et al. [8] found that the central part of the Vigo Ría became characterized by high contents of Fe and As. Selenium, contrarily, is widely used in the electronics industry, in TV cameras, in solar batteries, in computer cores, in rectifiers, in xerographic plates, and in ceramics as a colorant for glass, but it is also used as a trace element for animal feeds [31].

Manganese and iron are elements that naturally occur in the environment. The distribution of Mn concentrations is shown in Table 2. The concentration values of Mn in seawater samples in Vigo Ría ranged from 1.3 to 2.8 µg L^−1^. The maximum mean value of Mn (2.62 ± 0.19 µg L^−1^) was observed in the inner side of the estuary (east side), in comparison with the minimum mean value (1.83 ± 0.32 µg L^−1^) at the open sea (west side). The concentrations of iron in seawater samples in Vigo Ría ranged from 3.3 to 5.2 µg L^−1^. The maximum mean value of Fe (4.60 ± 0.46 µg L^−1^) was also observed in the inner side of the estuary (east side), in comparison with the minimum mean value (3.91 ± 0.69 µg L^−1^) at the open sea (west side). This shows that both elements are very influenced by river sources [5,32].

Therefore, considering aquatic life criteria from EPA [33], only Cu exceeds both the Criterion Maximum Concentration (CMC) for acute toxicology of 4.8 µg L^−1^ and the Criterion Continuous Concentration (CCC) for chronic toxicology of 3.1 µg L^−1^. Only 1 of 18 seawater samples (sample number 62, at the south-west side of the estuary) gave a higher concentration of 6.9 µg L^−1^ in the Carreira Bay (also known as Patos Bay), at the north of the small peninsula of Monteferro, which is the main ship entrance/exit to the estuary of Vigo along the coastline. Although this was a spot sampling, it is interesting to increase surveillance in the area. Compounds with copper in their structures, such as Cu_2_O (copper oxide) and CuCNS (copper thiocyanate), have been alternatively used as biocides to prevent encrustation of marine organisms on ships’ hulls [34].

Other usages of copper include electrical cabling and plating, copper piping, photo-graphy, anti-fouling paints, formulation of pesticides, and metal effluents from municipal wastes; the most industrial sources include manufacturing, refining, and coal-burning industries [35]. The concentration level of Cu among the sampling locations is shown in Table 2. The concentration values of Cu in seawater samples ranged from 0.7 to 6.9 µg L^−1^. The highest mean value of Cu (2.23 ± 1.83 µg L^−1^) was recorded in the south side of the estuary, by the city harbor of Vigo, in comparison with the minimum mean value (1.34 ± 0.74 µg L^−1^) at the north side of the estuary, next to the tourist beaches. The cited anthropogenic sources may lead to considerable concentrations entering the coastal and marine environments, either directly through discharged sewage or industrial effluents or via depositions from the atmosphere. Sánchez-Marín et al. [29] and Beiras et al. [36], after four sampling campaigns, showed the evolution of three points at Cangas, Moaña, and Vigo urban areas from clean to moderately polluted, and two points located in the industrial Vigo harbor can be considered heavily polluted.

Zinc is one of the most abundant and movable of the heavy metals and is transported in natural waters in both dissolved forms and attendant with suspended fragments [37]. The concentration of Zn in seawater surface ranged from 0.2 to 10.3 µg L^−1^. The highest average concentration of Zn (1.71 ± 3.23 µg L^−1^) in seawater was observed by the Vigo city harbor (south side), in comparison with the minimum mean value (0.62 ± 0.41 µg L^−1^) at the north side of the estuary, next to the tourist beaches. It was found that Cu and Zn concentrations were highly correlated between them (Figure 4), showing a similar pollution origin, probably due to anti-fouling paints [38], in such a way that, by linear regression analysis between Cu or Cu/Fe ratio and the rest of elements or element ratios, the following highly significant equations were found:

Cu = 0.577 × Zn + 0.101691 × Se (R^2^ = 92.96%) (1)

Cu/Fe = 0.575306 × Zn/Fe + 0.104448 × Se/Fe (R^2^ = 93.32%) (2)

In the comparison of Cu concentrations in different coastal areas (Table 3), the Vigo estuary has lower levels than the highly polluted areas of Xiangshan Bay (China; [39]) and the Yellow Sea (South Korea; [40]) and shows occasional higher copper levels than the Laoshan Bay (China; [41]) and the Gulf of Cádiz (South Spain; [42]).

## 4. Conclusions

The chemical distribution of ten dissolved trace metals and metalloids (Cr, Mn, Fe, Cu, Zn, As, Se, Cd, Hg, and Pb) in water was determined from north to south (where the biggest city in the region is) and from east to west (where the maritime traffic is higher). Metal concentration in waters showed that most metals were below recommended water quality criteria by EPA. The levels of heavy metals found only reflect the photo of the summer of 2018 in the Vigo estuary. The results showed that one of the samples had a Cu concentration higher at the entrance of the Vigo estuary, where maritime traffic is very important, especially in summer since there is high ferry boat traffic to the Cies Islands, as well as more boats and yachts of vacationers. It was found that Cu and Zn concentrations were highly correlated between them, showing a similar pollution origin, probably due to anti-fouling paints. It was also found that Mn and Fe are elements influenced by river sources.

## Figures and Tables

**Table 1:** Concentration range, calibration (a: intercept, b: slope, r^2^: determination coefficient), limits of detection (LOD), and quantification (LOQ), in µg L^−1^, for metals and metalloids analyzed in surface seawaters.

	^52^Cr	^55^Mn	^56^Fe	^63^Cu	^66^Zn	^75^As	^78^Se	^111^Cd	^202^Hg	^208^Pb
**Range**	0–50	0–50	0–100	0–100	0–500	0–50	0–100	0–50	0–5	0–50
**a**	0.0206	0.0105	0.6011	0.0181	0.0240	4.29 × 10^−4^	0.0216	1.43 × 10^−4^	0.0042	0.0231
**b**	0.1224	0.1118	0.1185	0.0795	0.0210	0.0195	0.0021	0.0244	0.0376	0.3589
**LOD**	0.050	0.012	0.155	0.084	0.294	0.050	2.548	0.009	0.350	0.007
**LOQ**	0.163	0.040	0.515	0.281	0.981	0.168	8.493	0.029	1.165	0.023
**r^2^**	0.9998	0.9996	0.9997	1.0	0.9990	0.9999	0.9997	0.9990	0.9999	0.9991

**Table 2:** Statistics (mean ± standard deviation, minimum, maximum, median ± interquartile range, and lower and upper quartiles) for elements detected (µg L^−1^) separated by cardinal points in the Vigo estuary.

Subzone	Statistics	Cr	Mn	Fe	Cu	Zn	As	Se	Cd	Pb
**North**	Mean ± SD	1.54 ± 0.18	2.13 ± 0.43	4.28 ± 0.56	1.34 ± 0.74	0.62 ± 0.41	4.90 ± 0.53	11.5 ± 3.9	0.01 ± 0.03	0.06 ± 0.10
	Min	1.4	1.5	3.3	0.7	ND	4.3	ND	ND	ND
	Max	2.0	2.8	5.2	3.2	1.6	5.7	17.2	0.1	0.3
	Median ± IR	1.50 ± 0	2.00 ± 0.40	4.40 ± 0.60	1.10 ± 0.40	0.50 ± 0.30	4.80 ± 1.00	12.3 ± 7.1	0 ± 0	0 ± 0.1
	Lower Q	1.5	1.9	4.0	0.9	ND	4.4	ND	ND	ND
	Upper Q	1.5	2.3	4.6	1.3	ND	5.4	15.2	ND	0.1
**South**	Mean ± SD	1.43 ± 0.12	2.06 ± 0.54	4.00 ± 0.81	2.23 ± 1.83	1.71 ± 3.23	4.76 ± 0.46	10.0 ± 2.4	0.01 ± 0.03	0.20 ± 0.36
	Min	1.2	1.3	2.7	0.8	ND	3.9	ND	ND	ND
	Max	1.6	2.8	5.5	6.9	10.3	5.5	12.7	0.1	1.1
	Median ± IR	1.50 ± 0.10	2.10 ± 0.80	4.10 ± 0.90	1.60 ± 1.00	0.60 ± 0.60	4.90 ± 0.50	10.0 ± 2.0	0 ± 0	0 ± 0.20
	Lower Q	1.4	1.7	3.6	1.3	ND	4.5	9.5	ND	ND
	Upper Q	1.5	2.5	4.5	2.3	1.0	5.0	11.5	ND	0.2
**East**	Mean ± SD	1.57 ± 0.22	2.62 ± 0.19	4.60 ± 0.46	1.73 ± 0.79	0.72 ± 0.51	5.00 ± 0.44	10.6 ± 3.7	0.03 ± 0.05	0.10 ± 0.13
	Min	1.4	2.3	4.2	0.9	ND	4.5	ND	ND	ND
	Max	2.0	2.8	5.5	3.2	1.6	5.5	15.6	0.1	0.3
	Median ± IR	1.50 ± 0.20	2.65 ± 0.30	4.50 ± 0.10	1.55 ± 0.60	0.60 ± 0.50	4.95 ± 0.90	11.3 ± 5.5	0 ± 0.10	0.05 ± 0.20
	Lower Q	1.4	2.5	4.4	1.3	ND	4.6	ND	ND	ND
	Upper Q	1.6	2.8	4.5	1.9	0.9	5.5	12.7	0.1	0.2
**West**	Mean ± SD	1.45 ± 0.11	1.83 ± 0.32	3.91 ± 0.69	1.82 ± 1.70	1.39 ± 2.82	4.74 ± 0.50	10.8 ± 3.2	ND	0.14 ± 0.32
	Min	1.2	1.3	2.7	0.7	ND	3.9	ND	ND	ND
	Max	1.6	2.3	5.2	6.9	10.3	5.7	17.2	ND	1.1
	Median ± IR	1.50 ± 0.10	1.85 ± 0.45	3.90 ± 0.90	1.25 ± 0.95	0.50 ± 0.45	4.75 ± 0.60	10.6 ± 3.7	ND	0 ± 0.10
	Lower Q	1.4	1.6	3.45	1.0	ND	4.4	8.65	ND	ND
	Upper Q	1.5	2.05	4.35	1.95	0.85	5.0	12.35	0	0.1
Saltwater CMC		1100			4.8	90	69	290	33	140
(Acute) (µg L^−1^)										
Saltwater CCC		50			3.1	81	36	71	7.9	5.6
(Chronic) (µg L^−1^)										

Underlined in grey, the significant highest (*p* = 0.05) average values. ND: not detected; CMC: criterion maximum concentration and CCC: criterion continuous concentration according to the United States Environmental Protection Agency (EPA).

**Table 3:** Concentration ranges (µg L^−1^) of dissolved heavy metals in different coastal areas of the world.

Region	Sampling year	Cr	Mn	Fe	Cu	Zn	As	Se	Cd	Pb	Reference
Laoshan Bay	2017–2018	0.31−2.71			0.51–4.50	0.09−5.71	0.63−1.75		0.059−0.769	0.16−9.13	[41]
(China)											
Xiangshan Bay (China)	2011–2016	ND–2.0			ND−44.5	0.7−65.9	0.6−8.5		0.01−1.61	0.06−8.08	[39]
Yellow Sea	2018	0.07−0.66			0.64−15.20	0.20−4.47	0.83−2.51		0.002−0.088	0.01−0.75	[40]
(South Korea)											
Kendari Bay	2014	0.085−0.386							0.001−0.015	0.009−0.549	[43]
(Indonesia)											
North Australian (coast and estuaries)	1996–2000			0.14−34.1	0.15−1.04	0.018−0.49	0.39−1.35		0.002−0.034	<0.002−0.057	[44]
Gulf of Trieste	2018–2019 *	<0.08−0.31	6.09−16.9	<0.9−3.51	0.41−1.74	6.93−31.2	2.10−2.31			<0.03−0.08	[45]
Adriatic Sea											
(Italy)											
Gulf of Cádiz^(1)^	2016			0.15−54.4	0.45−10.9	0.59−55.3			0.015−0.81	0.006−0.83	[42]
three estuaries											
(South Spain)											
Atlantic Ocean ^(1)^ (North and South)	1990						1.33−1.56	0.082−0.099			[46]
Portuguese Coast ^(1)^	2010				0.057−2.86	0.091−4.05			0.001−0.10	0.002−0.031	[47]
Ferrol Ría ^(1)^)	2000–2001				0.43−0.58	1.11−1.57			0.010−0.011	0.048−0.063	[48]
(Northwest Spain											
Vigo Ría ^(1)^	2002–2003				0.069−0.59	0.48−1.25			0.002−0.011	0.017−0.052	[19]
(Northwest Spain)											
Vigo Ría	2018	1.20−2.00	1.30−2.80	2.70−5.50	0.70−6.90	0.20–10.3	3.90−5.70	5.70−17.20	ND−0.100	ND−1.10	This study
(Northwest Spain)											

ND= not detected; * Since it was published in 2020, sampling is supposed to be in 2018–2019 (winter, summer, and autumn); (1) Transformed from nM into μg L^−1^ to keep the same units throughout the table. Underlined in light- or heavy-grey indicates increasing magnitude level.

## Data Availability

The data presented in this study are available in article or in suplementary files.

## Supplementary Materials

The following are available online at https://www.mdpi.com/1660-4601/18/4/2136/s1, Table S1: Latitude (Lat), longitude (Long) and spatial quadrant of sampling points, Table S2: Metal contents in a synthetic seawater enriched with most of the elements quantified.

## Abbreviations

EPA: United States Environmental Protection Agency, ICP−MS: inductively coupled plasma—mass spectrometry, LOD: limit of detection, LOQ: limit of quantification, ANOVA: analysis of variance, PCA: principal component analysis, ND: not detected, CMC: criterion maximum concentration, CCC: criterion continuous concentration.
