# Supplementary material for: Coastline Levels of Dissolved Heavy Metals in the Estuarine Water–System of Vigo
Source: Int J Environ Res Public Health. 2021 Feb 22;18(4):2136. doi: 10.3390/ijerph18042136 (PMC7926957; doi:10.3390/ijerph18042136)
Supplement: Supplementary file 1 [file ijerph-18-02136-s001.pdf]

Supplementary Table S1.

Latitude (Lat), longitude (Long) and spatial quadrant of sampling points.

Sample	Lat	Long	Quadrant*
**VG053**	42,2744167	-8,6874	NE
**VG054**	42,2552833	-8,73731667	NE
**VG055**	42,2389333	-8,78563333	NE
**VG056**	42,2392333	-8,8269	NW
**VG057**	42,2420333	-8,8668	NW
**VG058**	42,2230333	-8,8668	NW
**VG061**	42,18295	-8,85068333	SW
**VG062**	42,176255	-8,83627	SW
**VG063**	42,1371183	-8,83942333	SW
**VG069**	42,2474317	-8,744365	SE
**VG079**	42,13828	-8,857767	SW
**VG081**	42,20005	-8,8507	SW
**VG089**	42,27157	-8,700947	NE
**VG090**	42,2379333	-8,78773333	NE
**VG091**	42,23685	-8,78771667	NE
**VG093**	42,2416833	-8,86743333	NW
**VG098**	42,2548833	-8,71041667	SE
**VG099**	42,2537833	-8,70835	SE

* The study area was divided into 4 quadrants (southwest –SW–, northwest –NW–, southeast –SE– and northeast –NE–) centered on the geometric center of the sampled area.

Supplementary Table S2.

Metal contents in a synthetic seawater enriched with most of the elements quantified.

**Element**	**Spiked content ($\mu g\ L^{-1}$)**	**Found content ($\mu g\ L^{-1}$)**	**Recovery (%)**	$\boldsymbol{t}_{\mathbf{value}}$*
**^{52}Cr**	20.58	19.81 ± 0.37	96.26	3.604
**^{63}Cu**	23.25	21.70 ± 2.17	93.33	1.237
**^{66}Zn**	21.52	22.01 ±. 2.27	102.2	0.794
**^{75}As**	22.66	23.10 ± 1.77	101.9	1.468
**^{111}Cd**	20.17	18.62 ± 0.68	92.31	3.948
**^{202}Hg**	17.47	17.04 ± 1.82	97.56	1.095
**^{208}Pb**	17.55	17.66 ± 1.64	100.6	0.116

All data are expressed as average value and standard deviation of three determinations.
*$t_{critical}$= 4.303 (p= 0.05)
